# Evaluation of multi-hazard map produced using MaxEnt machine learning technique

**DOI:** 10.1038/s41598-021-85862-7

**Published:** 2021-03-22

**Authors:** Narges Javidan, Ataollah Kavian, Hamid Reza Pourghasemi, Christian Conoscenti, Zeinab Jafarian, Jesús Rodrigo-Comino

**Affiliations:** 1 Department of Watershed Management, Faculty of Natural Resources, Sari Agricultural Sciences and Natural Resources University (SANRU), Sari, 48441-74111 Iran; 2 Department of Natural Resources and Environmental Engineering, College of Agriculture, Shiraz University, Shiraz, 71441- 65186 Iran; 3 Department of Earth and Marine Sciences (DISTEM), University of Palermo, Palermo, 90123 Italy; 4 Department of Range Management, Sari Agricultural Sciences and Natural Resources University (SANRU), Sari, 48441-74111 Iran; 5 Department of Physical Geography, University of Trier, 54296 Trier, Germany; 6 Soil Erosion and Degradation Research Group, Department of Geography, Valencia University, Blasco Ibàñez, 28, 46010 Valencia, Spain

**Keywords:** Environmental sciences, Natural hazards

## Abstract

Natural hazards are diverse and uneven in time and space, therefore, understanding its complexity is key to save human lives and conserve natural ecosystems. Reducing the outputs obtained after each modelling analysis is key to present the results for stakeholders, land managers and policymakers. So, the main goal of this survey was to present a method to synthesize three natural hazards in one multi-hazard map and its evaluation for hazard management and land use planning. To test this methodology, we took as study area the Gorganrood Watershed, located in the Golestan Province (Iran). First, an inventory map of three different types of hazards including flood, landslides, and gullies was prepared using field surveys and different official reports. To generate the susceptibility maps, a total of 17 geo-environmental factors were selected as predictors using the MaxEnt (Maximum Entropy) machine learning technique. The accuracy of the predictive models was evaluated by drawing receiver operating characteristic-ROC curves and calculating the area under the ROC curve-AUCROC. The MaxEnt model not only implemented superbly in the degree of fitting, but also obtained significant results in predictive performance. Variables importance of the three studied types of hazards showed that river density, distance from streams, and elevation were the most important factors for flood, respectively. Lithological units, elevation, and annual mean rainfall were relevant for detecting landslides. On the other hand, annual mean rainfall, elevation, and lithological units were used for gully erosion mapping in this study area. Finally, by combining the flood, landslides, and gully erosion susceptibility maps, an integrated multi-hazard map was created. The results demonstrated that 60% of the area is subjected to hazards, reaching a proportion of landslides up to 21.2% in the whole territory. We conclude that using this type of multi-hazard map may be a useful tool for local administrators to identify areas susceptible to hazards at large scales as we demonstrated in this research.

## Introduction

Natural disasters are serious threats to human life and properties all over the world. Preventing natural catastrophes is not conceivable, yet by developing suitable preparation plans and mitigation measures its drawbacks can be alleviated^1^. Considerable morphological changes in landforms due to active tectonics or climate changes can impact control in human activities^2–9^. However, also humans can drastically modify natural ecosystems, negatively. For example, deforestation, non-sustainable agricultural management or human-made constructions can increase soil mobilization and the transportation of sediments, resulting in extreme land degradation processes^10–13^. Combining land degradation processes allow us understanding environmental issues and threats difficult to be assessed because of its complexity.

Events such as gully erosion, landslides, and floods are physical phenomena, active in geological times but uneven in time and space^14–17^. They are considered hazard events, which can be induced by humans or not, but all of them are key global issues threatening human life, resources and goods^18–21^. Moreover, they have different drawbacks in various places and because of their correlated subsequences, these catastrophes have contrary long-term effects. When these penalties have a considerable impact on human life and activities, they become natural disasters^23,24^. Since human interventions in natural ecosystems that cause natural catastrophe to lead to endangering human life and significant economic consequences, the awareness of society is vital to reduce them^25–27^. Mitigating the effects of potential catastrophes and preparing the proper infrastructure for tackling them requires notably accurate information about the vulnerability and susceptibility of a specific territory about environmental hazards^22^. In general, natural disasters occur more frequently overpassing the human capability to restore the effects of past events^28^. Hence, it is necessary to plan and manage the natural catastrophes to decrease both the economic penalties and loss of humankind life. To achieve this goal, it is key to consider the natural catastrophe predictive maps throughout the land use planning stages.

Since natural hazards are difficult to be predicted, most of the studies focus on a single hazard to be mapped. However, unfortunately, it is usual that several hazards occur at the same time in one place. Therefore, there is a necessity to make integrate studies although they are more complex and difficult to be represented in one synthesized map or article. During the last years, major developments have been done to quantify the feedback, mechanisms and interconnection among different hazards and factors^29–31^. One of the most relevant achievements are the multi-hazard mapping initiative (MMI), which started by the Federal Emergency Management Agency (FEMA) to provide multi-hazard advisory maps^32,33^ and the novel UN framework for catastrophe risk decrement sturdily highlights the necessity of a multi-hazard approach^34^.

These models allow showing the spatial pattern of a natural phenomenon, environmental elements, or some human activities, and provide information on the spatial distribution of natural hazards such as flood, landslides, and erosion and they are important tools for planners and environmental managers to identify susceptible areas and to prioritize their mitigation response efforts^28,35–38^. A multi-hazard susceptibility map (MHSM) represents susceptibility and hazard information, together, on a single combined map. Because of the large number of maps and their probable variance in the area covered by different scales, applying a single hazard map to supply information on every single hazard, is complicated for planners^39^. Alternatively, a MHSM raised from the synthesis of different hazard maps would allow giving proper information from a particular area and it could help the land planners to analyze all of them from a holistic point of view. The multi-hazard map is an accurate tool to create awareness in mitigating multiple hazards^40^ and also for the selection of appropriate land uses and evaluation of susceptibility areas. By the way, the United Nation has emphasized the significance of multi-hazard assessment and referred that it “is an essential element of a safer world in the twenty-first century”. Nevertheless, analyzing a multi-hazard map is complicated and requires major challenges as well as the analysis of susceptibility^41^.

In this regard, several types of research have focused on multi-hazard evaluation via GIS-based methods that make it possible to analyze various data and the improvement of natural hazard models for a specific area^41–44^. For example, it is representative the research conducted by Sheikh et al. in the Golestan province at a large scale to assess a multi-hazard-based management using a coupled TOPSIS–Mahalanobis distance^45^. Also, there are several inventive, statistical, and deterministic methods that can be used in a single hazard or even multi-hazards^28,46–56^. Data-mining models have newly been suggested with predictive skills and progressive pattern learning and can present a good platform to synthesize and to analyze the information for the definition of potential hazard areas^57–63^. The most common methods proposed in the literature are artificial neural networks^64,65^, frequency ratio-FR^17,66^, logistic regression^67^, index-of-entropy^68^, fuzzy logic^69^, and multivariate adaptive regression splines^31,70^. Also, the development of machine learning and statistical methods techniques, including support vector machine (SVM), random forest (RF), boosted regression trees (BRT), maximum entropy (MaxEnt) has contributed significantly to the field of natural hazards^71–73^. Among these, maximum entropy has been successfully used for assessing different types of natural hazards such as landslides^55,67^, floods^74^, gully erosion^75^ and soil salinity^76^ due to fast and easy implementation and robust mathematical functions and theoretical backgrounds.

So, the main aims of the current research are: (1) to explore the ability of the MaxEnt model to predict the spatial occurrence of flood, landslides, and gully erosion; (2) to better understand the relationships between these processes and their controlling factors; and (3) to design a methodological perspective for preparing a combined multi-hazard map for land use planning and hazard mitigation. To achieve these goals, we present a study case in the Gorganrood Watershed, which has witnessed several landslides, gully erosion, and floods that have been a matter of debate in recent years^77,78^.

## Study area

**Figure 1:**
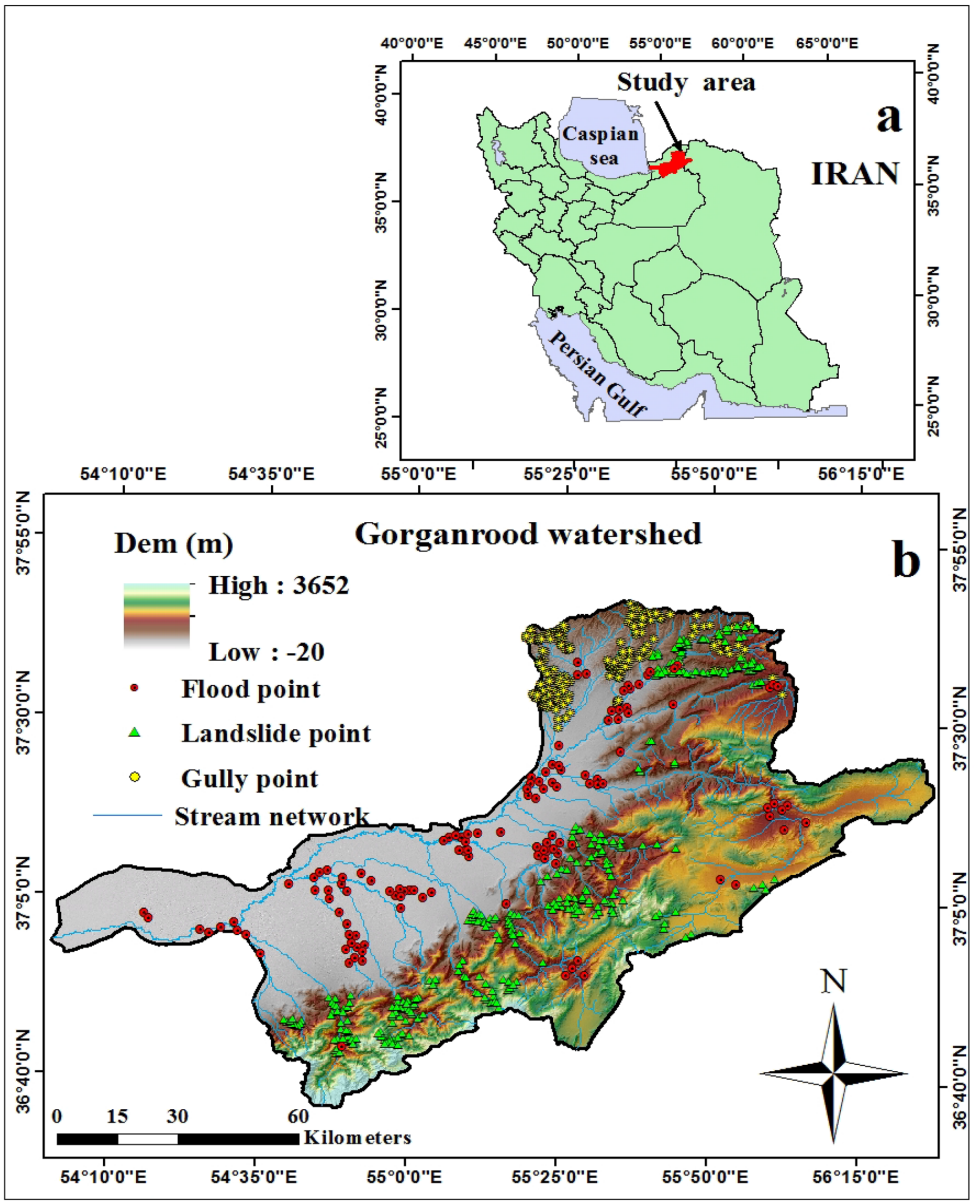
Location of the study area, sampling points and elevation.

The Gorganrood Watershed is located in the Golestan Province which is situated in the north-eastern part of Iran and covers an area of 10,197 km^2^. The study area lies between the latitudes of 36° 34′ to 38° 15′ N and the longitudes of 54° 5′ to 56° 8′ E (Fig. 1). Topographically, it is characterized by steep slopes, up to 69° in mountainous regions. The central and western parts are generally characterized as plain and flat areas with an average elevation of between 95 and 3652 m^78^. The annual mean rainfall is approximately 231–848 mm^78^. The southern section has a typical mountain climate and the central and northern regions have a Mediterranean climate. The average minimum and maximum temperatures are 11 and 18.5 °C, respectively^80^. In the last decade, this area has been challenged with different natural hazards such as erosion, landslides, and floods which was selected as an appropriate application site for the multi-hazard probability assessment (MHPA).

**Figure 2:**
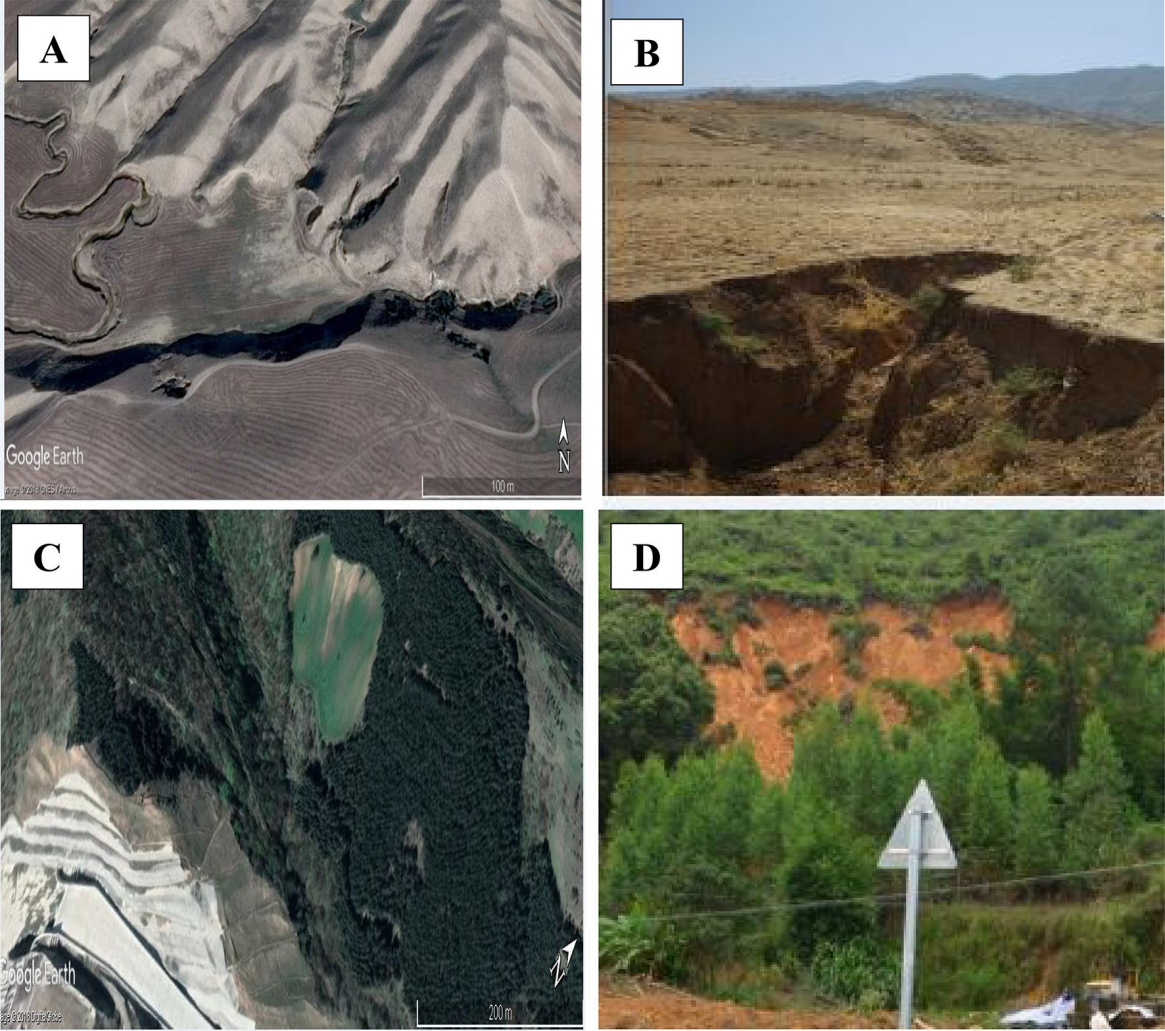
Some examples of gully erosion (a, b) and landslides (c, d) in the Golestan Province, Iran. *(**A**) and (**C**) were obtained from Google Earth, and (**B**) and (**D**) were taken by Narges Javidan.

According to national reports, the study area is covered with some prone lithological formations such as dark grey shale, sandstone, and Quaternary deposits^78^. Regarding the infrastructures, the area consisted of main cities, villages, and 1218 km of national roads, where to some extent, they can be exposed to a divertimento of natural hazard occurrences^78^. Figure 2 presents some photographs of gully erosion locations (a, b), landslides (c, d), and flood (e) in Golestan Province.

In this area, one of the deadliest flash floods occurred on 10th August 2001 which caused up to 300 people deceased, 381 harmed, and 4000 buildings were endured heavy loss^79^. Moreover, 99 mm^3^ (millions square meters) of sediments loaded behind the dams, and also made damages to rangelands, forests, and residential units^78^. Some authors estimated 430,000 hectares affected by erosion because of gullies, landslides, etc. from 1990 to 2005. In this province, about 5–6 t/ha/year soil erodes in forest areas^80^. Also, because of the existence of the steep slopes in the study area, the landslide is one of the destructive events in the area and often destroys gardens and agricultural land, the damage of roads and natural resources^55^. Loss of soil, the imposition of plenty of costs, reduced agricultural potential and has caused the migration of people in the villages of this region.

## Methodology

**Figure 3:**
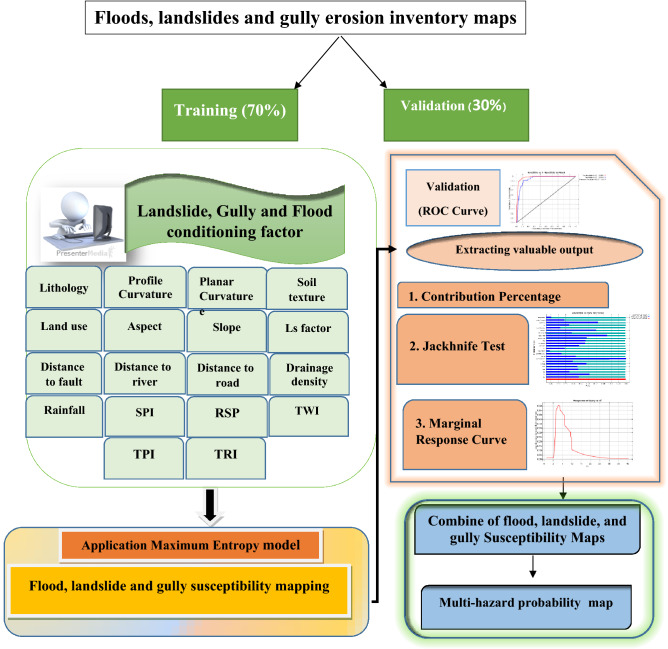
Flowchart of the methodology used for the MHPM in Gorganrood Watershed, Golestan Province, Iran. *Own elaboration.

Figure 3 illustrates the methodological flowchart of this approach that was used for the MHPM analysis using the MaxEnt model. The flowchart comprises main four steps: (1): preparing thematic layers (17 geo-environmental conditioning factor); (2): gully erosion, flood, and landslides susceptibility modelling using the ME machine learning techniques; (3): validation of the susceptibility maps using the ROC-AUC curve; and, (4) to combine flood, landslides, and gully erosion susceptibility maps to prepare a multi-hazard probability map for land use planning and hazard mitigation.

### Landslides, gully erosion, and flood inventory mapping

A key step for susceptibility mapping is the preparation of an inventory of hazard landforms^81^. The landslide, gully erosion, and flood inventory for the Gorganrood Watershed were compiled from field investigation and national and regional documents from various organizations including the Water Resources Organization and Department of Natural Resources Management of Golestan. Considering that some hazard locations are located in mountainous areas and the field investigation may miss them, Google Earth images were used for landslides identification, as well. The inventory map for gully erosion is a collection of occurrences (283 gully locations), where a landslide inventory map is containing 351 landslide locations. Some authors highlighted that using analysis of the past documents of flood occurrences, the future flood events in an area can be estimated^82^. So, in this study, a flood inventory map was prepared by containing 127 flood locations. In general, a random partition algorithm^83,84^ was used to separate training points from the validation points. In the current study, 70% of each hazard was used in the model building (training) and the remaining 30% were used for the validation. Three replications and three sample data sets were used to perform these processes including S1, S2, and S3. These datasets were arranged to evaluate the robustness of the built models and data sensitivity^81,85,86^. The equal data sets (positives/negatives) are applied which includes all the positive cells (hazard locations) and the same randomly inferred negative cells (non-hazard locations).

### Flood, landslides, and gully erosion conditioning factors

It is essential to determine the effective factors on different natural hazards and human-made fatalities to performing flood, landslides, and gully erosion susceptibility maps, separately^87^. A good understanding of the main hazards-related factors is needed to recognize the susceptible areas. For this aim, the conditioning factors for different hazards were selected from the literature review^55,59,84,88,89^. In this study, ArcGIS 10.5 (ESRI, USA) and System for Automated Geoscientific Analyses (SAGA) software were used to produce and display these data layers. For the application of the MaxEnt machine learning model, all the factors were converted to a raster grid with 30 × 30 m grid cells. Entire the conditioning factors were primarily continuous, and some of them were classified within different categories based on expert knowledge and literature review^36,90–92^.

As in Suppl. Material 1, the predicting factors used in this study for three different types of hazards are as follows: (a) Digital Elevation Model/elevation (m), (b) slope aspect, (c) slope per cent, (d) land use, (e) plan curvature, (f) profile curvature, (g) TWI, (h) lithological units (i) drainage density (mm), (j) soil texture, (k) distance to streams (m), (l) annual mean rainfall, (m) relative slope position, and for Landslide are (n) distance to faults (m), (o) stream power index, (p) LS factor Also (q) distance to roads (m) was employed for gully erosion. Table 2 also shows the predicting factors used in this work for three hazards.

DEM (Digital Elevation Model) of the subject area with a 30 m pixel size was produced using digital contours data prepared from the Department of Natural Resources Management of Iran (Suppl. Material 1 a-c). From this DEM, some geomorphological layers such as slope^93–96^, hillslope aspect^97–102^, curvature layers^103,104^ were obtained using ArcGIS 10.5 software (ESRI, USA). The slope curvature map was compiled with three categories: convex, concave, and flat. Positive curvature exhibits convex (> + 0.1), negative curvature depicts concave (< − 0.1), and zero curvature represents flat (− 0.1 to  + 0.1). Also, profile and plan curvatures possess a range of positive and negative values and return a different description in every single index. Positive and negative values in profile curvature demonstrate convexity (increasing flow velocity) and concavity (reducing flow velocity), respectively. On the contrary, positive and negative values in the plan curvature denote concavity (flow convergence) and the convexity (flow divergence), respectively^54,105^. Values close to zero represent neutral curvature in both cases.

Land use/land cover (Suppl. Material 1 d) plays a significant role in the operation of hydrological and geomorphological processes by directly or indirectly influences on evapotranspiration, infiltration, run-off generation, and sediment dynamics^102,106^. The land use/land cover map of the subject area in 1:100,000-scale was prepared from the Natural Resources Office of Golestan Province and modified by Google Earth images. The land use/ land cover of the subject area comprises of the lake, residential areas, forest lands, rangelands, drying farming, irrigation farming, rocky lands, and saline lands. Soil texture is generally recognized as a weighty controlling factor in the mechanism of infiltration and runoff generation and is effective on hazard occurrence^107–109^.

This layer was created by digitizing the soil texture map of Golestan Province (1:100,000-scale) gained from the Agriculture Department, Iran. The soil texture in the subject area comprises of sandy-loam, clay-loam, sandy-clay-loam, silty-clay, silty-clay-loam, and silty-loam (Suppl. Material 1 g). The topographic position index (TPI) approach was applied to assess topographic slope location, and to zone ordination automation, which creates a single-band raster characterized quantities measured upon elevation^110^. It is an algorithm increasingly applied to measure topographic slope positions and displays the corresponding position of each cell (Suppl. Material 1 h).

Moore and Grayson^111^ and Grabs et al.^112^ mentioned that TWI (Topographic wetness index) represents the tendency of gravitational forces and the spatial distribution of wetness conditions to move water to the downslope. This factor has been prepared using Eq. (2):

1 $$TWI=ln\left(\frac{\propto }{tan\beta }\right)$$

where *α* is the cumulative upslope area draining through a point (per unit contour length) and tan β is the slope angle at the point. In this survey, the TWI map was prepared in SAGA-GIS and its value ranges from 1.20 to 22.92 (Suppl. Material 1 i).

Distance to streams is one of the key conditioning factors due to its importance on the flood magnitude and spread of landslides and gully erosion^48,113^. Layers of the proximity were produced using the Euclidean distance function in ArcGIS 10.5 software and varying from 0 to 11,720 m for roads (Suppl. Material 1 r), 0–15,080 m for streams (Suppl. Material 1 j), and 0–55,212 m for faults (Suppl. Material 1 s). The roads and rivers were derived from the national topographic map at the scale of 1:50,000 whereas faults extracted from geology map in 1:100,000-scale. Based on field studies, landslides are distributed typically nearby the linear features especially faults and roads. Landslide hazard level is closely related to the proximity to faults and roads. It affects not only surface structures but also terrain permeability^100,101,114^. Where water flow concentrates may be appropriate for hosting gullies, road construction undoubtedly has a sturdy negative impact on slope stability^115^. The drainage density (Suppl. Material 1 k) is also one of the main conditioning factors that strongly contribute to many hazards’ occurrence^59^. According to Tehrany et al.^88^, a high drainage density causes a larger surface runoff ratio. The drainage pattern of a region is influenced by different factors such as the structure and nature of the soil characteristics, geological formation, infiltration rate, slope degree, and vegetation cover condition^83^. To convert the drainage network pattern to measurable quantity, the drainage density was determined using an extension of” line density” in ArcGIS 10.5 software. Rainfall-triggered landslides have brought great damages to communication sub-structures, properties, and pasture biomass production^116–118^. The annual mean rainfall map of Gorganrood Watershed was prepared based on the rainfall data extracted from the Regional Water Organization of Golestan Province. This map created using fifty-three stations and a statistical period of 2001–2016 based on the Inverse Distance Weight (IDW) interpolation method (Eq. 3). This map ranges from 384 to 810 mm/year. The rainfall map was prepared in a raster format of 30 × 30 m in ArcGIS 10.5 as an input layer for assessment of hazard (Suppl. Material  1 l).

2 $$\mathrm{\lambda i}=\frac{{\mathrm{Di}}^{-\mathrm{\alpha }}}{\sum_{\mathrm{i}=1}^{\mathrm{n}}{\mathrm{Di}}^{-\mathrm{\alpha }}}$$

where *λi* is the weight of the point *i*, Di is the distance between the points *i* and the point of the unknown, and *α* is equal to the weighing power^119^. Assuming that discharge is associated with the specific catchment area, the erosive power of water flow can be measured by the stream power index-SPI (Suppl. Material 1 m)^111^:

3 $$SPI=As\times tan\sigma$$

where *As* represents the specific catchment area in meters and *r* is the slope gradient in degrees. The SPI index is one of the most important factors controlling slope erosion processes since the erosive power of running water straightly influences river cutting and slope toe erosion^120^. The areas with high stream power indices have an excessive potential for erosion because it is representative of the potential energy procurable to entrain sediment^121^. The relative slope position (RSP), as a tool, could calculate several terrain indices from the digital elevation model (Suppl. Material 1 n). General information on the computational concept can be found in^122^. The discrepancy between the value of one cell and the average value of the 8 surrounding cells defines the TRI (Terrain Ruggedness Index)^51^. In the first place, the two input neighborhood raster (using a 3 × 3 neighborhood for min and max) was produced from a DEM, afterwards, the equation was run in Raster Calculator (Suppl. Material 1 o). Lithological units (Suppl. Material 1 p) play a dominant role in determining gully erosion and landslides in each area^99,102,123,124^ because gully erosion is particularly dependent on the lithology properties and various lithological units demonstrate important differences in landslide instability. Also, lithology is assumed as a necessary factor in the spatial and temporal variations of drainage basin hydrology^125^. Lithological units have different susceptibility to active hydrological processes. In this study, the lithological map of the subject area was produced according to the available geological maps on a scale of 1:100,000 obtained from the Geological Survey Department, Iran. Different variety of lithological formations have covered the Gorganrood Watershed which is classified into 24 groups (Table 1).

**Table 1:** Litology of the Gorganrood Watershed.

Group	Code	Formation
1	Ksr	Sarcheshmeh
2	Pz1a.bv	–
3	E1c	–
4	kl	Ruteh
5	Jch	Chamanbid
6	Pr	Ruteh limestone
7	TRJs	Shemshak
8	Cm	Mobarak
9	Cl	Lalun
10	PlQc	Kashfrud
11	Sn	Niur
12	Jmz	Mozduran
13	ksn	Sanganeh
14	Murmg	Dalichai
15	Qft	Alluvial terrace
16	Jl	Lar
17	Dp	Khoshyeylaq
18	pCmt2	Padeha
19	Ekh	Khangiran
20	Kat	Aitamir
21	Pd	Dorud
22	Jbash	Bashcalateh
23	Qsw	–
24	TRe	Elikah

The LS (slope-length) plays as a significant factor in soil erosion and natural hazards occurrence^122^ and is known as a parameter used in the RUSLE equation to consider the effect of topography on erosion^126^. The topographical factor depends on the slope steepness factor (S) and the slope length factor (L) and was estimated based on the slope and specific catchment area as follow^127^:

4 $$LS={\left(As /22.13\right)}^{0.4}\times {\left(sin\beta /0.0896\right)}^{1.3}$$

where *As* is the specific catchment area and $\beta$ is the slope in degrees. The LS factor map for Gorganrood Watershed (Suppl. Material 1 q) was extracted using the SAGA-GIS software^122^.

### Multi-collinearity test

The factors were used to consider the effect of correlation among them as the independent variables (Table 2). When the correlation between two independent variables is considerably high, it is a problem in the modelling process. The problem is named multi-collinearity. The VIF (variance inflation factor) and tolerance are two significant indices for multicollinearity diagnosis. VIF is the reciprocal of tolerance, contrarily, tolerance is 1 − R^2^ for the regression of that variable in contradiction of all the other independents, deprived of the dependent variable^128^. A VIF of > 5 or 10 and more and/or tolerance of lower than 0.10 shows a multicollinearity obstacle^129,130^.

**Table 2:** Predicting factors for the three selected hazards in the study area.

Flood predicting factors	Landslide predicting factors	Gully predicting factors
Elevation (m)	Elevation (m)	Elevation (m)
Slope aspect	Slope aspect	Slope aspect
Slope percent	Slope percent	Slope percent
Land use	Land use	Land use
Plan curvature	Plan curvature	Plan curvature
Profile curvature	Profile curvature	Profile curvature
Topographic wetness index	Topographic wetness index	Topographic wetness index
Lithological units	Lithological units	Lithological units
Drainage density	Drainage density	Drainage density
Soil texture	Soil texture	Soil texture
Distance to streams (m)	Distance to streams (m)	Distance to streams (m)
Annual mean rainfall (mm)	Annual mean rainfall (mm)	Annual mean rainfall (mm)
Relative slope position	Relative slope position	Relative slope position
Terrain Ruggedness Index	Terrain Ruggedness Index	Terrain Ruggedness Index
Stream power index	Stream power index	Stream power index
	Distance to faults (m)	Distance to faults (m)
–	LS factor	LS factor
–	Distance to roads (m)	Distance to roads (m)

### Maximum entropy (MaxEnt) model

The MaxEnt model was applied in the Maxent software for modelling landslides, flood, and gully erosion and calculation of hazard values (version 13.0.6.0). Phillips et al.^131^ proposed the MaxEnt model for predictive modeling of geographical species distribution based on the most important environmental condition when presence data are available^131,132^. We can also explain the maximum entropy estimation from a decision-theoretic viewpoint as a sturdy Bayes estimation. MaxEnt depends on a machine learning response that makes predictions from incomplete data^133,134^. The MaxEnt output produces in ASCII format as a continuous prediction of specific presence that ranges from 0 to 1^134^. For running the MaxEnt model, validation and training datasets were processed in excel format, and the conditioning factors were converted from raster to ASCII format, which is needed in Maxent software^135^. During the model running, for model training in the calibration phase, a random selection algorithm was used and (70%) of datasets were randomly selected^83^. This machine learning technique allows for the investigation of the relationship amongst a dependent variable (landslides, flood, and gully occurrence) and several independent variables (conditioning factors/geo-environmental factors), respectively. Details are given in Phillips et al.^132^.

### Considering the effect of variables importance using the MaxEnt model

In the current study, the sensitivity analyses^136,137^ has been used as an exploratory technique to define the effect of variable variations on model outputs, allowing then a quantitative evaluation of the relative importance of uncertainty sources. To assess the uncertainty of projected maps in this study, a Jackknife test was executed for investigative the effects of removing any of the conditioning factors on the three susceptibility maps^138^. The Jackknife test can be used to assess the relative strengths of every predictor variable^131,138,139^. In consonance with the Jackknife test outcomes, variables with zero values including (SPI and soil for flood modelling; SPI, LS, profile curvature, and aspect for gully erosion; and TWI for landslides) were eliminated. Therefore, the remaining variables were used to run the final model for all three hazards.

### Evaluation of the predictive performance of three hazards models

The validation step is the most important process of modelling^140^. The prediction accuracy of the built hazard models was evaluated by the ROC curve. In this approach, the AUC can evaluate the prediction accuracy qualitatively^119,141^. The ROC curve is a methodical technique that has been using to describe the proficiency of deterministic and probabilistic and prediction systems^142^.

The prediction accuracy of the models based on the AUC value can be classified three classes of accuracy following the classification proposed by Hosmer & Lemeshow^143^: 0.7, 0.8, and 0.9 *AUC* value thresholds were adopted to acceptable, excellent, and outstanding performance, respectively^81,89^.

### The multi-hazard mapping adoption process

The combination of three hazard maps was used to create the multi-hazard probability map including flood, gully erosion, and landslide. First of all, for every considered hazard in this study, the MaxEnt model was constructed. Afterwards, the multi-hazard probability map was prepared based on the three individual hazard susceptibility maps, by synthesizing the three individual susceptibility maps according to their four classes in ArcGIS 10.5 environment, and this multi-hazard susceptibility map was ultimately classified into eight classes.

## Results

### Results of the multi-collinearity test

According to the results of Table 3a,b,c, TRI (Terrain Ruggedness Index) for landslides, flood, and gully erosion with VIF > 5 and tolerance < 0.1 was eliminated. So, other factors are used for future analyses, and results show there is not any multi-collinearity among the remaining independent variables in the present study.

**Table 3:** The results of multi-collinearity test.

Predictors	Collinearity Statistics	
	Tolerance	VIF
**(a) For flood predictors**		
Drainage density	0.386	2.589
Relative slope position	0.694	1.442
Slope	0.210	4.752
Soil	0.788	1.269
Stream power index	0.734	1.362
Topographic wetness index	0.656	1.525
Elevation	0.454	2.205
Distance to stream	0.428	2.335
Land use	0.902	1.109
lithology	0.687	1.455
Plan curvature	0.634	1.576
Profile curvature	0.658	1.519
Annual mean rainfall	0.547	1.828
Aspect	0.917	1.091
**(b) For landslide predictors**		
Plan curvature	0.729	1.372
Annual mean rainfall	0.610	1.641
Drainage density	0.334	2.997
Relative slope position	0.328	1.592
Slope percent	0.394	2.538
Soil texture	0.880	1.136
Stream power index	0.241	4.156
Elevation	0.451	2.215
Distance to fault	0.804	1.244
Distance to stream	0.311	3.217
Land use	0.857	1.167
Lithology	0.907	1.103
LS factor	0.444	2.250
Topographic wetness index	0.494	2.022
Profile curvature	0.604	1.657
Aspect	0.985	1.016
**(c) For gully erosion predictors**		
Plan curvature	0.639	1.564
Annual mean rainfall	0.580	1.723
Drainage density	0.385	2.598
Relative slope position	0.616	1.624
Slope percent	0.358	2.793
Soil texture	0.772	1.296
Stream power index	0.400	2.500
Topographic Wetness Index	0.557	1.796
Aspect	0.943	1.060
Elevation	0.427	2.342
Distance to road	0.733	1.364
Distance to stream	0.393	2.543
Land use	0.803	1.245
Lithology	0.818	1.222
Profile curvature	0.582	1.719

### Application of the MaxEnt model

**Figure 4:**
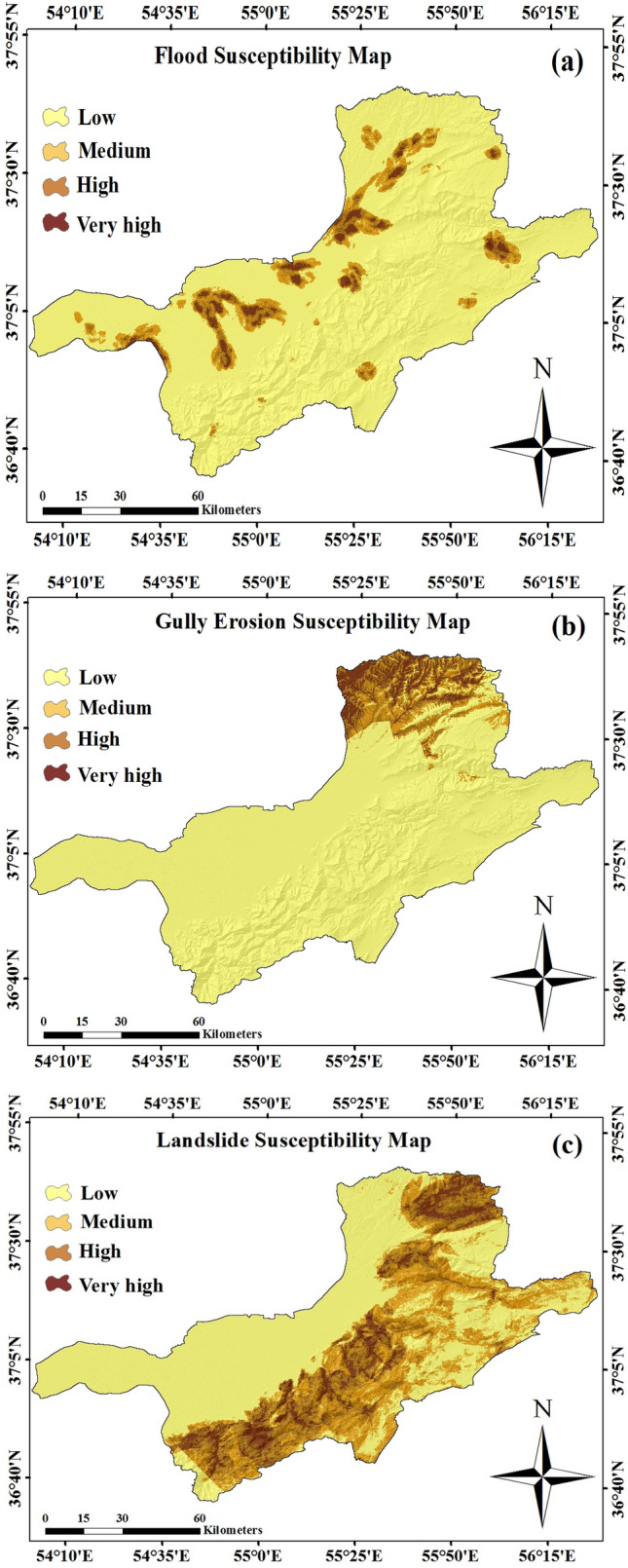
Susceptibility mapping for (**a**) floods, (**b**) gully erosion, (**c**) and landslides using MaxEnt model in the study area.

The susceptibility map for each hazard and each dataset in the study area was produced using both continuous and categorical data sets. Finally, the MaxEnt model was built using all three training groups of the sample data sets (i.e., S1, S2, S3) in the training step. Susceptibility maps of the flood, gully erosion and landslides of the study area are presented in Fig. 4. Four susceptibility groups including low (L), Moderate (M), high (H), and very high (VH) are performed based on the output susceptibility maps using the most authentic natural breaks classifying method.

**Figure 5:**
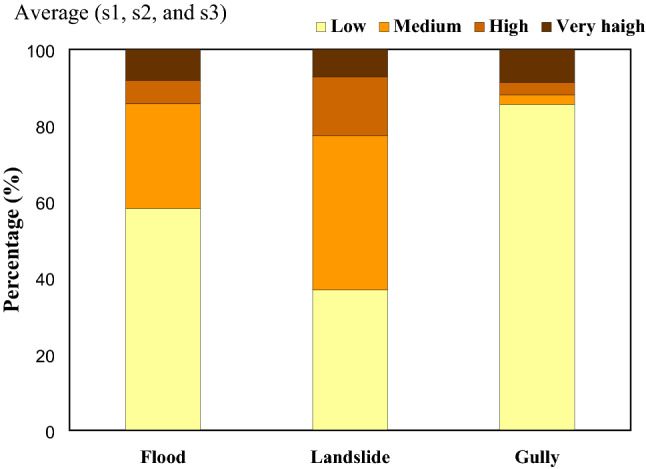
Relative distributions of the average of four susceptibility classes for the flood, landslides, and gully erosion susceptibility maps.

Also, Fig. 5 shows the relative distribution of the average of the flood, landslides, and gully erosion susceptibility classes for three categories of the sample data sets. Besides, the statistical characteristics of the probabilistic prediction of the three hazards and all sample data sets are shown in Table 4.

**Table 4:** Statistical characteristics of the probability values obtained from ME models.

Model	Data set	Probabilistic prediction values	
		Mean	SD
Flood	S1	0.0388	0.1184
	S2	0.0385	0.1151
	S3	0.0383	0.1186
Landslide	S1	0.1422	0.1882
	S2	0.1466	0.1879
	S3	0.1470	0.1884
Gully	S1	0.0437	0.1213
	S2	0.0449	0.1234
	S3	0.0472	0. 1284

*SD* Standard deviation.

### Sensitivity and response curves analysis

A sensitivity analysis^134,139^ was performed to investigate the relative strengths of every predictor variable on the results of predicted maps using the Jackknife test. Suppl. Material 2 shows the results of the Kappa-based Jackknife test using AUC on test data (S1) for flood, landslides, and gully erosion.

Suppl. Material 3 illustrates the response curves of one data set (S1) for some of the important conditioning factors used for three hazards (landslides, flood, and gully erosion) assessment.

### The MaxEnt model performance

**Figure 6:**
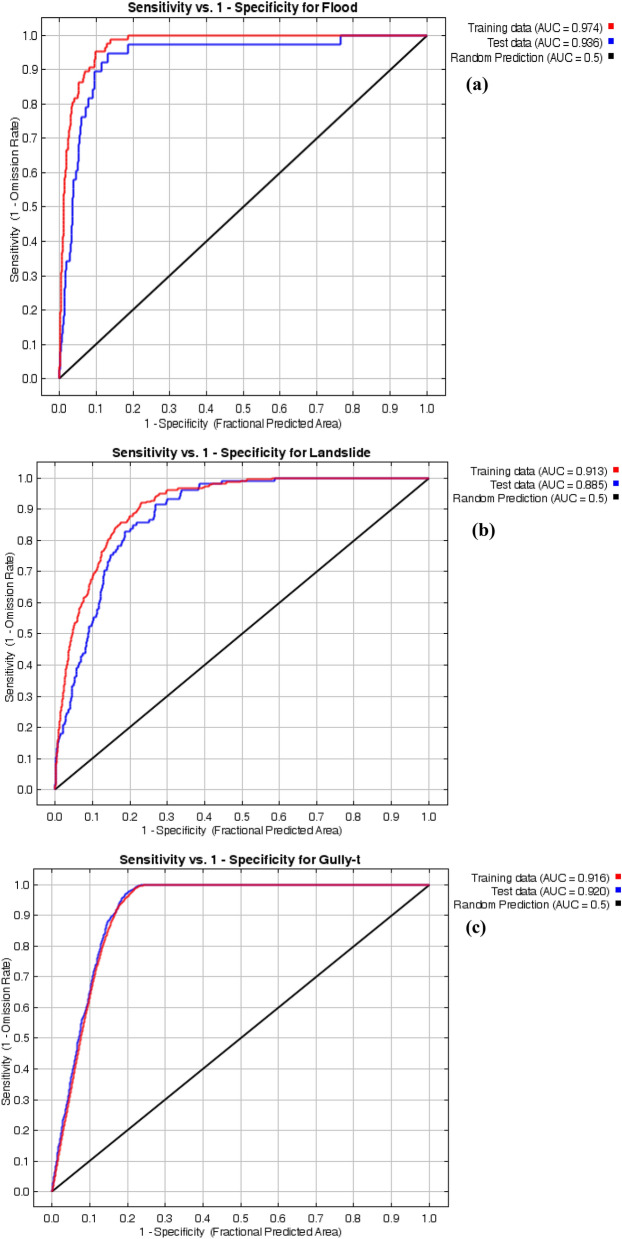
ROC curves of one data set (S1) for three hazards (**a**) landslides, (**b**) floods, and (**c**) gullies.

**Figure 7:**
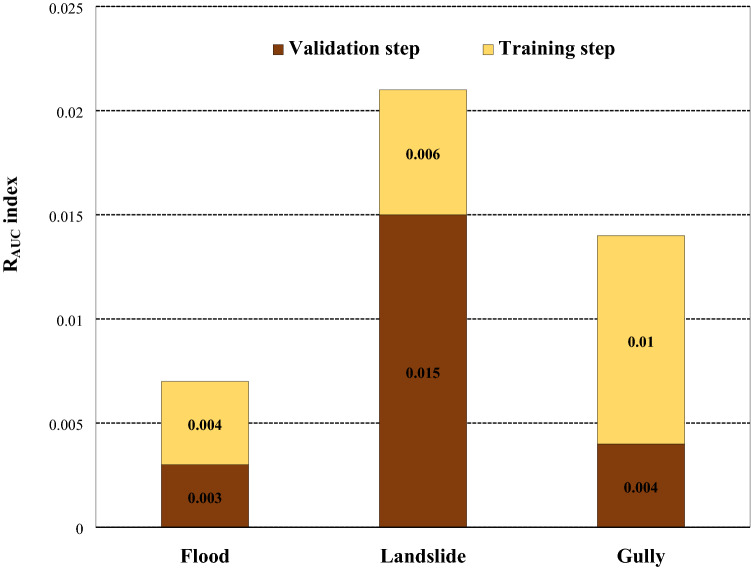
Robustness of the MaxEnt model in training and validation steps based on AUC.

The results of the MaxEnt model (based on all three sample data sets) show different ranges of susceptibility values of hazards. The results of the goodness-of-fit are shown in Table 5. Figure 6a–c show the AUROC value for the three forecasted hazards maps based on one data set. The hazards samples applied to the model evaluation must be different from the hazards points used for training. In this current work, 30% of hazards occurrence points (30% of floods, landslides, and gully erosion samples) were considered to the validation phase (Table 5). The outcomes of robustness according to AUROC are illustrated in Fig. 7.

**Table 5:** Predictive performance of models based on three sample data sets (S1, S2, and S3) in the training and validation step.

Evaluation criteria (AUC)	Data set	ME model		
		Flood	Landslide	Gully
Training	S1	0.974	0.913	0.916
	S2	0.974	0.914	0.920
	S3	0.970	0.919	0.926
	Mean	0.972	0.915	0.920
Validation	S1	0.936	0.885	0.920
	S2	0.933	0.883	0.918
	S3	0.936	0.870	0.922
	Mean	0.935	0.879	0.92

### Multi-hazard probability map (MHPM)

The individual probability maps (i.e., gully erosion, floods, and landslides) which created using the MaxEnt model were used to produce the multi-hazard susceptibility map by synthesizing the three various hazard maps and finally classified into eight different classes: landslides-gully-flood, landslides-floods, landslide-gully, gully erosion, floods, gully-floods, landslides, gully-floods, and no hazard.

**Figure 8:**
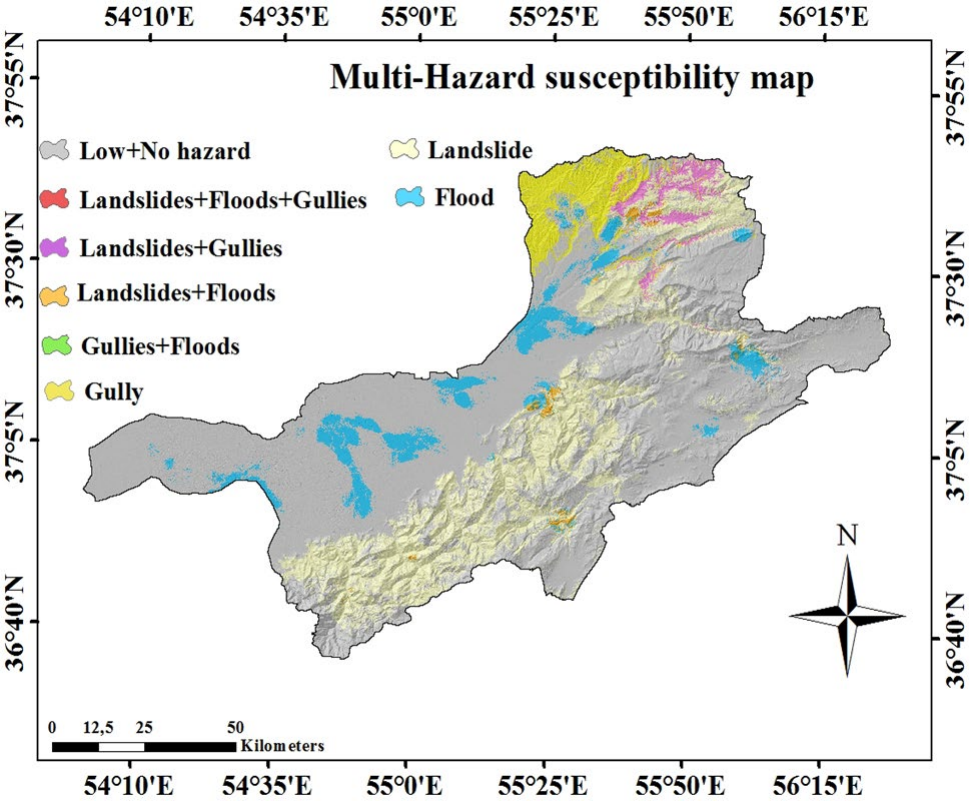
Multi-hazard probability map (MHPM).

**Figure 9:**
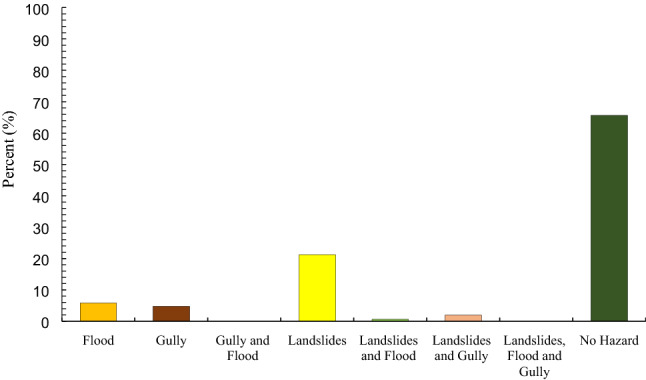
Percent of association of each hazard in the MHPM.

Figure 8 shows MHPM of the Gorganrood Watershed for the three hazards. Results demonstrated that 40% of the area is located in the low to very low susceptibility zones whereas 60% of the area is subjected to floods, landslides, and gully occurrence. It is also cleared that the proportion of landslide is the most occupied hazard (21.2%) in the Gorganrood Watershed (i.e. the range of areas covered by a landslide was larger than other hazards) (Fig. 9).

## Discussion

### Mapping hazards using combined diverse multi-risks

In our current study, after applying the multi-collinearity test, the susceptibility map for each hazard and each dataset was generated using an independent variable. Four susceptibility groups are performed based on the output susceptibility maps using the most authentic natural breaks classifying method^47,144^.

As Fig. 6 shows, for flood susceptibility mapping according to the MaxEnt model, less than 6% of the study area has a high and very high susceptibility, whereas, about landslides, approximately 14.8% and 8.1% of the study area was classified as high and very high classes, respectively. For the gully erosion susceptibility map, 8.2% of the pixels in the study area fell into high and very high susceptibility classes. For three hazard modelling, the highest percentage belongs to the low class.

Two techniques of one-by-one predictor-removal (OOPR) and only-one-predictor-involved (OOPI) considering the Jackknife-test were used to identify the key hazard-predictors. When isolated, the most influential predictor variables are drainage density, distance to streams, and DEM/elevation, respectively for flood, DEM/elevation, lithological units and annual mean rainfall landslides, and annual mean rainfall, DEM/elevation, lithological units for gully erosion. In other words, the elevation variable was the main controlling factor among all other variables for three hazards, whilst the lithological units were identified as the most important independent variable for gully erosion and landslides.

Therefore, as stated by Convertino et al.^145^, the SA (sensitivity analysis) allows modelers and managers to identify the conditioning factors (i.e. input variables) that reduce the variance of the model output to the most, which is significantly vital in understanding the model structure.

According to Suppl. Material 3, in the response curve of drainage density, with increasing river density, the flood values increased drastically, the most floods occurred in the range of drainage density between 0.3 and 0.6 km/km^2^. In other words, flood susceptibility increased in the areas with very high drainage density (i.e. increase the runoff transport capacity of drainage network). Based on the outcomes of previous studies, when the drainage density is high, it leads to an important surface runoff ratio. Different factors affect the drainage pattern of an area including slope degree, infiltration rate, vegetation cover condition the structure and nature of the soil characteristics, and geological formation^83^.

Regarding the distance to streams, the flood happened very close to rivers due to an upsurge in the degree of flood susceptibility. It is one of the important predictor factors owing to its significance on the flood velocity and magnitude. The high concentration of flow in the places around the stream network, there is the most chance of flood incidence close the streams^113^. Several studies highlighted that elevation, drainage density, and distance to streams were the most significant predictors for flood occurrence^103,145,146^.

The flood values decreased in places with high elevation. This was based on the outcomes from response curves of elevation, which demonstrates that flood happens in areas by low elevation and plains. In the areas with low elevation, an abundant amount of water enters the stream network and results in a flood incident^147^. The natural treatment of flooding, which happens mostly in flat areas instead of in highly elevated regions can produce a suitable proof for current results. Correspondingly, based on^148^, these areas have more upslope contributing area and runoff production. The most landslide occurrences happened in the range of elevation between 300 and 500 m, in which steep slope areas are located. The elevation did not contribute straightly to landslide appearance, but other factors such as precipitation and erosion processes were registered, both of them, logically affected by the elevation, therefore, relevant to be considered too^149^. As mentioned by other authors^150^, lithological unit structures and attributes constitute fundamental factors in landslide events. Various lithological units based on their types and characteristics have dissimilar landslide possibilities. In response curves of lithological units to landslide, the most hazard happened in group 21 with Dorud Formation, red sandstone, and shale with subordinate sandy limestone.

The underground hydrostatic level and water pressure surge because of rainfall^151^. One of the significant operating prognosticators in landslide mapping is landslide initiation which is powerfully connected with rainfall^151,152^. According to the results, the maximum percentage of the landslide is centralized in areas with rainfall rages from 650 to 690 mm. The gully extension hazard will rise in the high amounts of precipitation^153^. The response curves of rainfall to gully demonstrates the maximum amount of gully erosion that happened in a rainfall ranged from 450 to 500 mm.

Moreover, the elevation has an essential role in the spatial alteration of hydrological conditions for example runoff production rate, soil moisture, surface flow, and slope stability^63^. Based on the survey of the regions with elevation beneath 200 m and flat areas, our results show that they are more prone to this type of erosion, which can be ascribed to the vegetation cover^154^. Consequently, the area with high elevation showed a lower possibility of gully erosion incidence.

Lithological units are considered the most influential prognosticators regarding the gully occurrence^155^. The reason was that the parent materials have different hydraulic conductivity and shear stability. In this study, the gully *v* within the groups 1 and 13 lithological units with Sanganeh and Sarcheshmeh formations registered this issue, respectively.

Consequently, a relatively higher contribution susceptibility prediction was obtained among some categorical data sets. However, these lesser contributions of some categorical layers did not mean that the categorical data layers were unusable for susceptibility mapping. As discussed in a previous research^139,156^, all these categorical layers did affect the final prediction result. Then simultaneously considered with continuous data sets.

The results of the goodness-of-fit in Table 5 confirm that performance values for the applied models, based on the AUC-ROC case of flood range from 0.970 to 0.974 (average = 0.972), for landslides, vary from 0.913 to 0.919 (average = 0.915), and for gully erosion, the minimum value of AUC-ROC is 0.916 and the maximum one 0.926 (average = 0.920). Consequently, a high proficiency was acquired for all the three natural hazards studied in this research.

In this current work, 30% of hazards occurrence points were considered to the validation phase (Table 5). Outcomes of the MaxEnt model demonstrates that the AUROC ranges between 0.933–0.936 (average = 0.935) for the floods. In the case of the landslides, AUROC values vary from 0.870 to 0.885 (average = 0.879), whereas for gully erosion changes from 0.918 to 0.922 (average = 0.920). There is a powerful settlement among the output hazard maps of the MaxEnt model and the distribution of hazards occurrence points. According to Hosmer & Lemeshow^143^, the MaxEnt model revealed excellent performances for all datasets^81,89^. Hence, based on the estimated AUROC value, the employed model detected reasonable prediction proficiency in forecasting the hazard spatial potentiality map. Considering that the accuracy values are almost identical when the data sets change, there were only a few changes and the model for three hazards was robust and entirely stable.

The outcomes of robustness according to AUROC are illustrated in Fig. 7. As can be observed, the MaxEnt model for landslide had a maximum robustness value (0.015) in the validation step indicating the minimum stability and robustness in comparison with other hazards (0.004 and 0.003). Furthermore, from a model stability viewpoint, the almost excellent agreement between training and validation *AUC* values for the applied model demonstrates that this model is most stable and over-fitting has also been avoided^157^. Based on this result, it is obvious that the MaxEnt model can be applied as an efficient machine learning technique in susceptibility assessment for flood, landslides, and gully erosion.

These results are consistent with the study of other authors^75,157–160^, who intended for preparing susceptibility mapping of the natural disasters.

In this study, we concluded that the MaxEnt is useful to model natural hazards (e.g. flood, landslides, and gully erosion occurrence) with nonlinear relationships. This machine learning model does not need a prior elimination of outliers or data transformation and can fit complex nonlinear relationships between hazards conditioning factors and hazards susceptibility. Also, it is able to automatically analyze interaction effects among conditioning factors (i.e. predictors)^54^. Our results demonstrated that 40% of the area is located in the low to very low susceptibility zones, whereas 60% of the area is subjected to floods, landslides, and gully occurrence. It is also clarified that the relevance of landslides is the most important (21.2%) in the Gorganrood Watershed (Fig. 9).

The most vulnerable areas for human activities are the ones that are in a group of more than one hazard. The flat area with low elevation are more prone to gully erosion and flood in the Gorganrood watershed whilst higher elevation areas with a high slope degree are more susceptible to landslides.

In a similar study, Pourghasemi et al.^160^ employed the SWARA-ANFIS-GWO model for producing a multi-hazard susceptibility mapping in Lorestan Province, most sections were susceptible to landslide and flood incidents together (33.7%), although 17.1% of the study region was in the class of no hazards. Nevertheless, in other researches, the various models have been employed for preparing multi-hazard susceptibility mapping. The surveyed hazards were floods, earthquakes, and landslides^160^, landslide, earthquake, and floods^161^, landslides, flood, and forest fire^162^, landslides, floods, earthquakes, forest fires, subsidence, and drought^163^, although there were several studies underway to develop multi-hazard risk^28,35–37^.

## Conclusions and final remarks

Using the MaxEnt machine learning technique in the Gorganrood Watershed, we generated individual flood, landslides, and gully erosion susceptibility maps and, subsequently, a combination of them, to estimate a multi-hazard probability map (MHPM). Results showed almost 40% of the area is placed in the low to very low susceptibility zones, but 60% of the area is subjected to floods, landslides, and gully occurrences. The proportion of landslide is the most common hazard in the Gorganrood Watershed (21.2%). Hazard and risk strategies should be considered before future occurrences. So, this research demonstrates the application of the MHPM may be utilized in other territories for land use planning and hazard mitigation giving new facilities for insurance purposes. To date, there are still no libraries of multi-hazard probability map available for three hazards used in the current study (i.e. flood, landslides, and gully erosion). A conformity procedure is needed to elaborate and design the mitigation practices, provided that individual accomplishments are harmonized with existing policies. Applying a great number of individual hazards maps with spatial covering and various resolutions for the planner is confusing. Therefore, an integrated multi-hazard probability map prepares homogenized data about frequent natural hazards for an area.

Risk and hazard management should be taken into account before studying disaster management. To begin such activities and schematization for further land use planning, the provided flood, gully erosion, and landslide susceptibility maps and the hazard-based probability mapping can be valuable platforms. Multi-hazard evaluation makes it possible reducing hazard risk and gives fundamental information for stakeholders, it can also provide a comprehensive vision of the changes happening in the environment. According to this point, a multi-hazard probability map can be applied for comprehensive and integrated land use planning and consequently for the watershed management.

## Supplementary information

Supplementary information.
