# Supplementary material for: Evaluation of multi-hazard map produced using MaxEnt machine learning technique
Source: Sci Rep. 2021 Mar 22;11:6496. doi: 10.1038/s41598-021-85862-7 (PMC7985520; doi:10.1038/s41598-021-85862-7)
Supplement: Supplementary file 1 — Supplementary information. [file 41598_2021_85862_MOESM1_ESM.docx]

**Evaluation of multi-hazard map produced using MaxEnt machine learning technique**

Narges Javidan^1^, Ataollah Kavian^1^, Hamid Reza Pourghasemi^2^, Christian Conoscenti^3^, Zeinab Jafarian^4^, Jesús Rodrigo-Comino^5,6^

^1^Department of Watershed Management, Faculty of Natural Resources, Sari Agricultural Sciences and Natural Resources University (SANRU), Sari 48441-74111, Iran; [narges.javidan20@gmail.com](mailto:narges.javidan20@gmail.com); a.kavian@sanru.ac.ir

^2^Department of Natural Resources and Environmental Engineering, College of Agriculture, Shiraz University, Shiraz 71441- 65186, Iran; hr.pourghasemi@shirazu.ac.ir

^3^Department of Earth and Marine Sciences (DISTEM), University of Palermo, Via Archirafi 22, 90123 Palermo, Italy; [christian.conoscenti@unipa.it](mailto:christian.conoscenti@unipa.it)

^4^Department of Range Management, Sari Agricultural Sciences and Natural Resources University (SANRU), Sari 48441-74111, Iran; [z.jafarian@sanru.ac.ir](mailto:z.jafarian@sanru.ac.ir)

^5^Department of Physical Geography, University of Trier, 54296 Trier, Germany

^6^Soil Erosion and Degradation Research Group, Department of Geography, Valencia University, Blasco Ibàñez, 28, 46010 Valencia, Spain. [jesus.rodrigo@uv.es](mailto:jesus.rodrigo@uv.es)

Corresponding authors: Ataollah Kavian ([a.kavian@sanru.ac.ir](mailto:a.kavian@sanru.ac.ir)) and Jesús Rodrigo-Comino ([jesus-rodrigo@uv.es](mailto:jesus-rodrigo@uv.es))


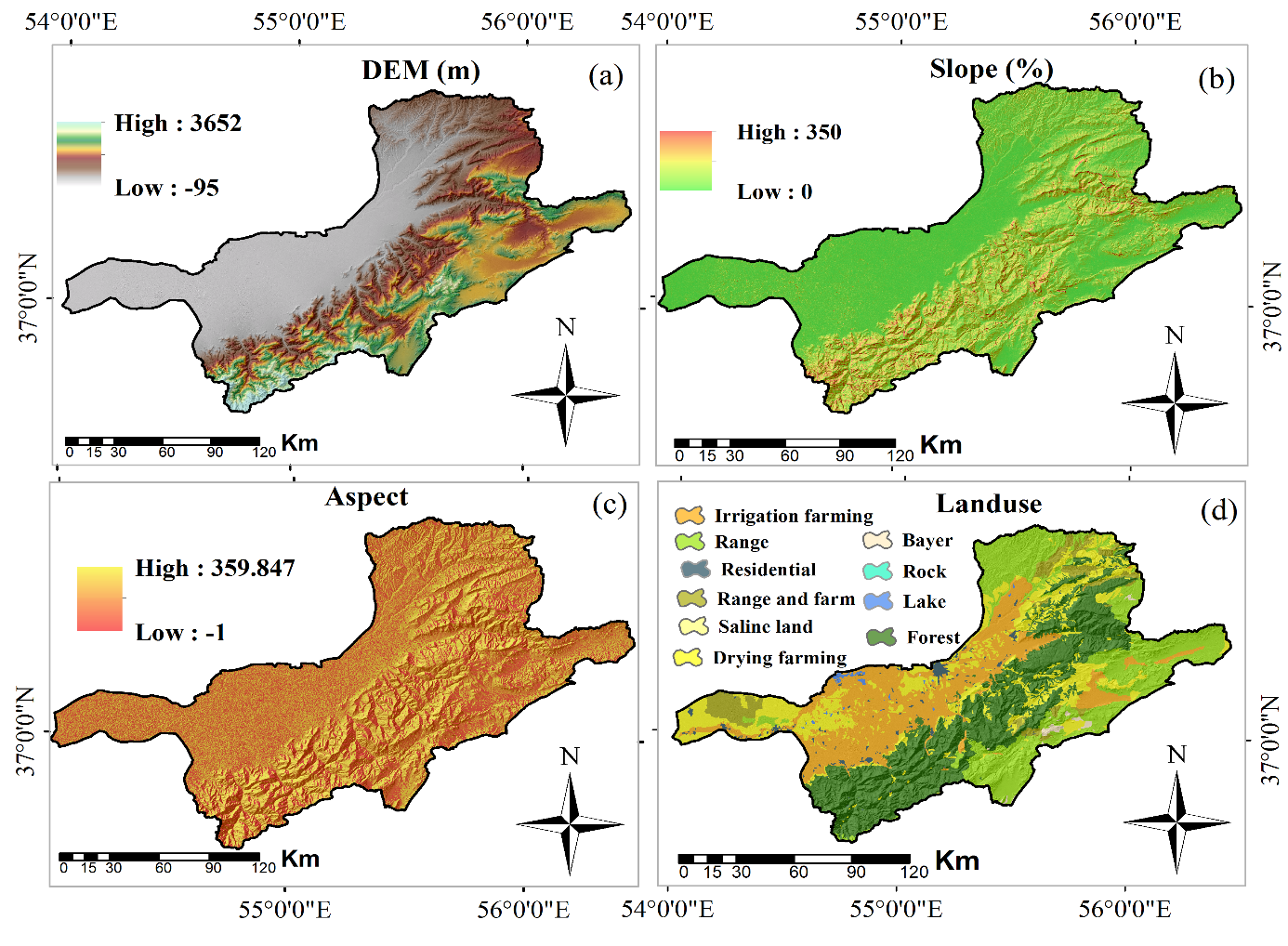


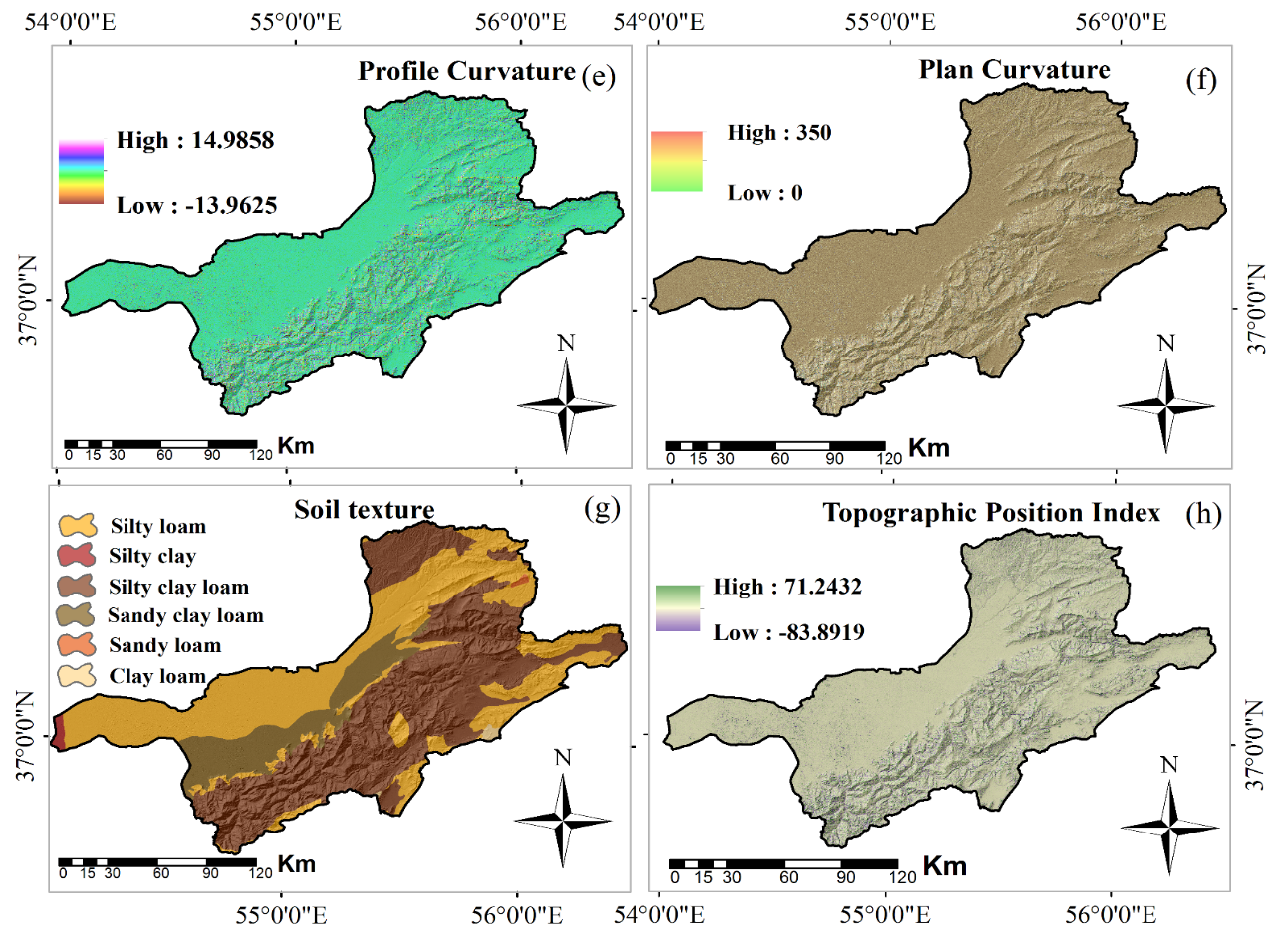


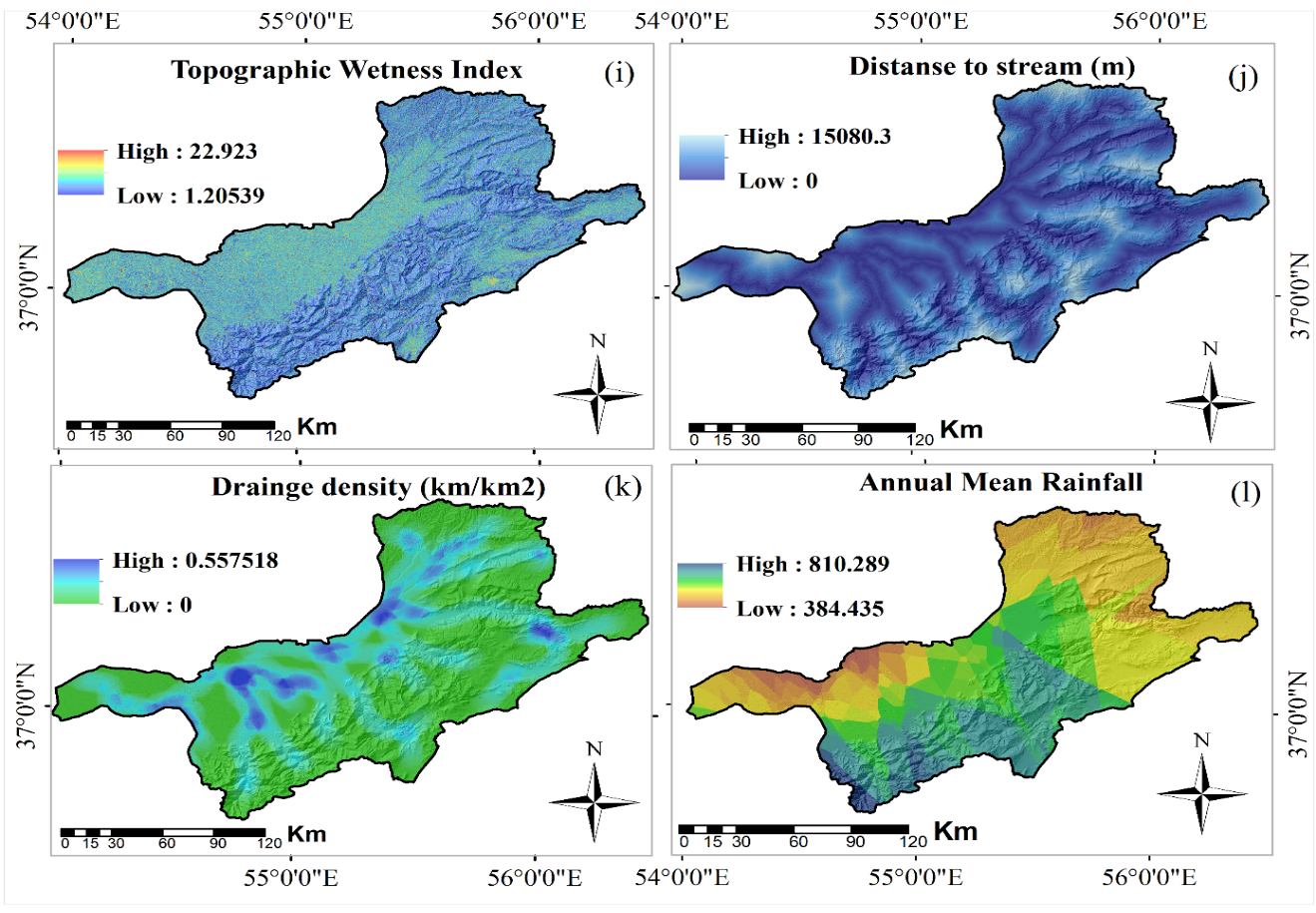


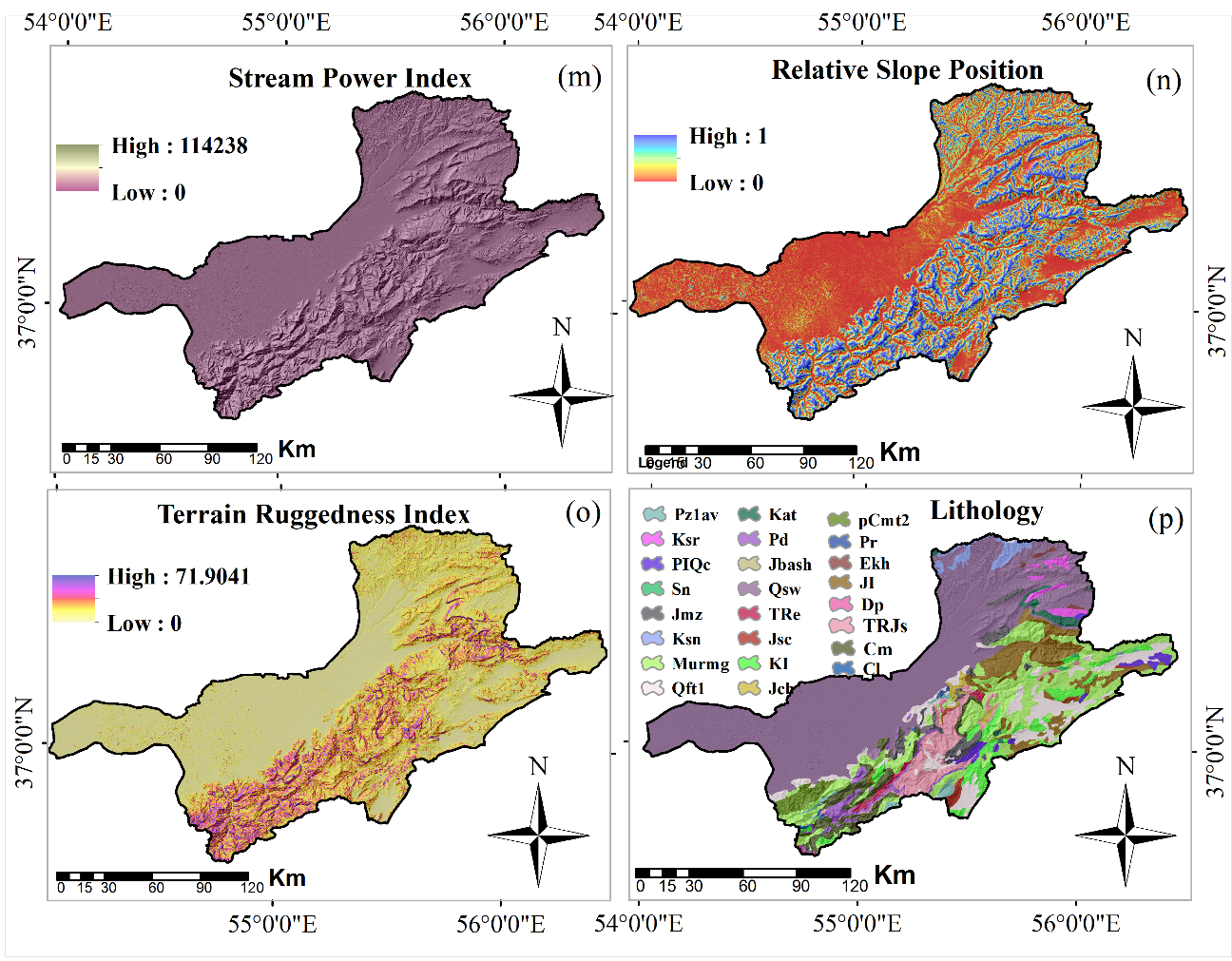


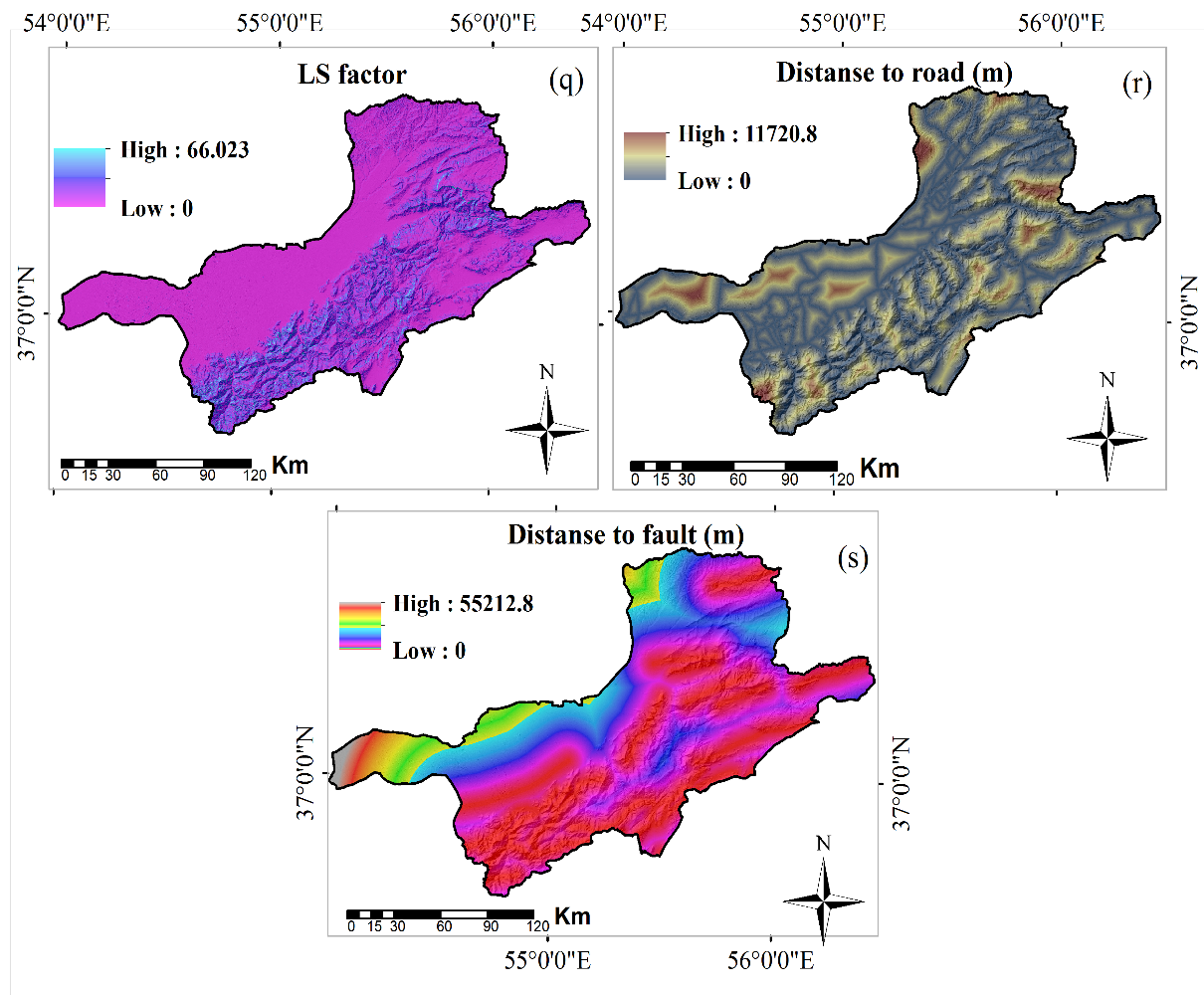


**Suppl. 1.** Maps displaying the flood, gully and landslide conditioning factors: **a)** DEM (m), **b)** Slope percent **c)** Slope aspect, **d)** Land use, **e)** Profile curvature, **f)** Plan curvature, **g)** Soil texture, **h)** Topographic Position Index (TPI) **i)** Topographic Wetness Index (TWI), **j)** Distance to streams (m), **k)** Drainage density (km/km^2^), **l)** Annual mean rainfall (mm), **m)** Stream Power Index, **n)** Relative Slope Position, **o)** Terrain Ruggedness Index, **p)** Lithology**, q)** LS factor**, r)** Distance to road (m)**, s)** Distance to faults (m).


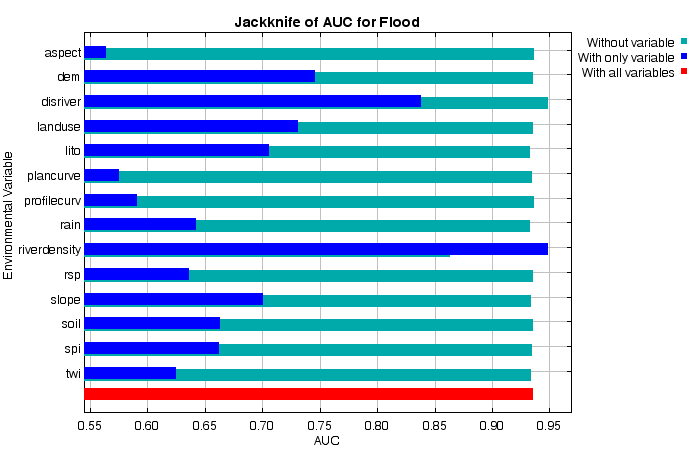


**(a)**


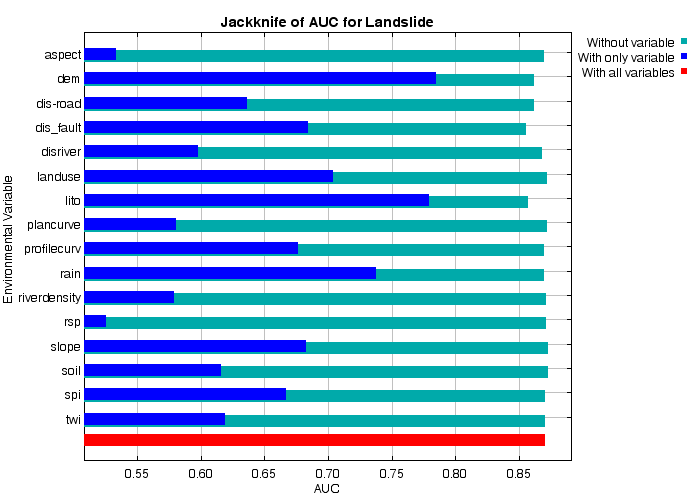


**(b)**


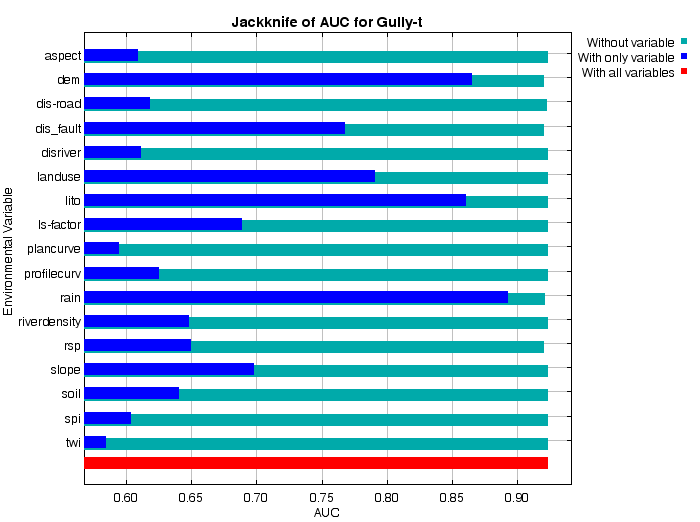


**(c)**

**Suppl. 2**. Factor importance analysis obtained from susceptibility model (**a)** Kappa-based Jackknife test for flood susceptibility, (**b)** Kappa-based Jackknife test for landslide susceptibility, and (**c)** Kappa-based Jackknife test for gully susceptibility.


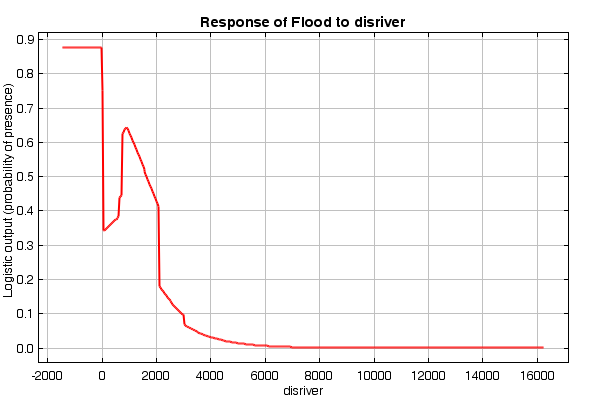

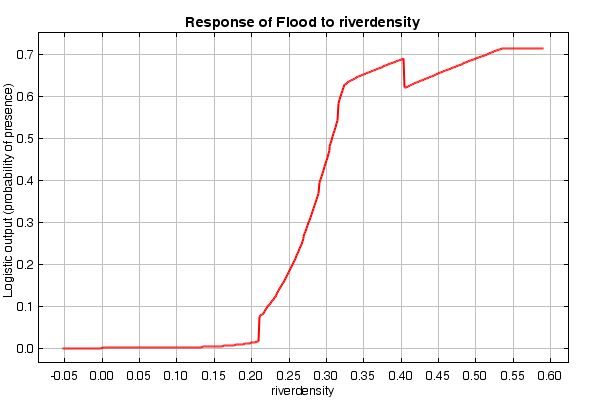


**(b)**

**(b)**

**(a)**


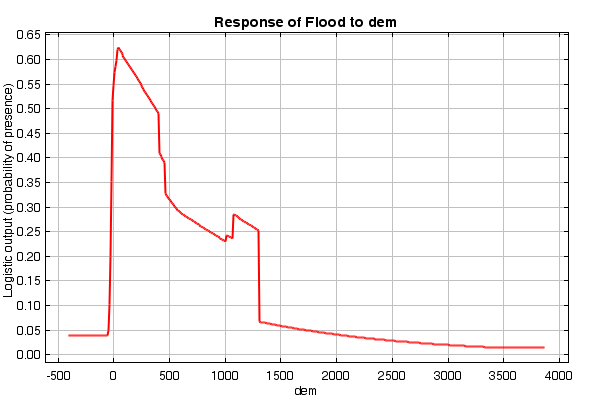


**(c)**


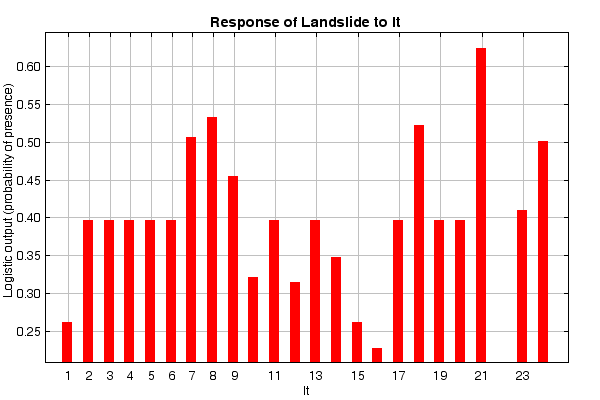

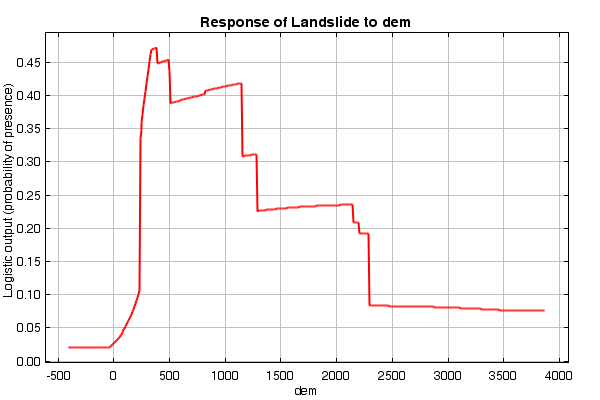


**(f)**

**(e)**

**(f)**


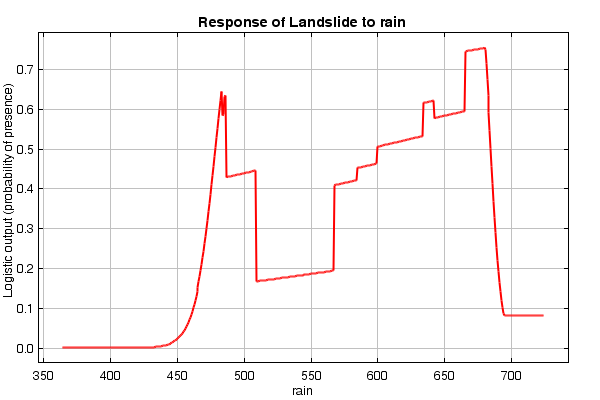


**(g)**


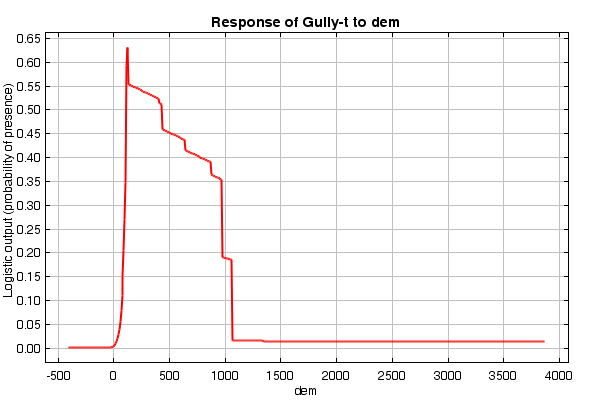

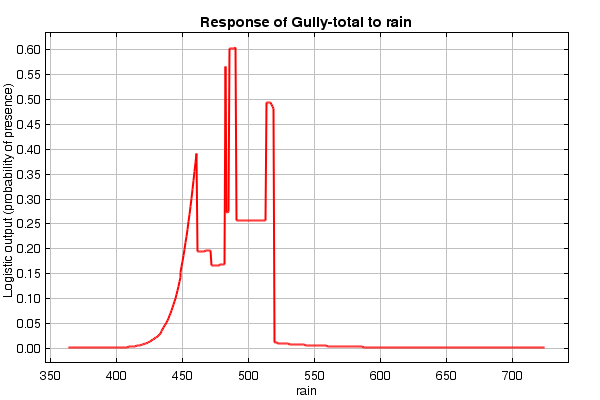


**(i)**

**(i)**

**(h)**


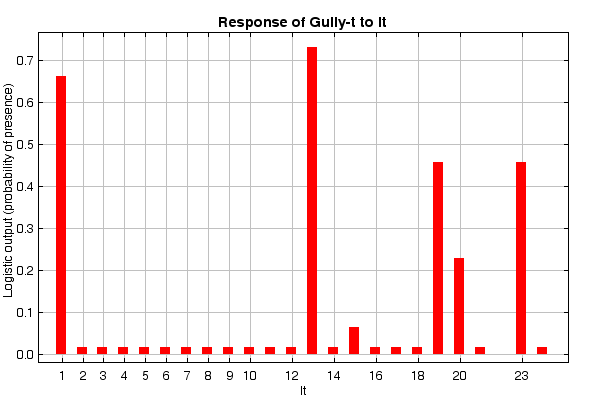


**(j)**

**Suppl. 3**. The response curves of one data set (S1) for some of important conditioning factors used for three hazards (a-b-c landslides, e-f-g flood, and h-i-j gully erosion) assessment
